# Short‐Term Puzzle Feeder Enrichment Increases Food Engagement but Not Stress‐Related Behaviour in Captive Golden‐Headed Lion Tamarins

**DOI:** 10.1002/ece3.73312

**Published:** 2026-03-27

**Authors:** Giulia Pipolo, Emma Chen, Elin Harlos, Johnny Möllerstrom, Juliano Morimoto

**Affiliations:** ^1^ School of Biological Sciences University of Aberdeen Aberdeen UK; ^2^ Tropikariet Helsingborg Sweden; ^3^ Institute of Mathematics University of Aberdeen, King's College Aberdeen UK; ^4^ Programa de Pós‐Graduação em Ecologia e Conservação Universidade Federal do Paraná Curitiba Brazil

**Keywords:** Callitrichidae, environmental enrichment, foraging behaviour, grooming, primate welfare, stress behaviour

## Abstract

Environmental enrichment is widely used in zoos to improve welfare and facilitate successful reintroductions, yet evidence for its effectiveness in callitrichids – particularly golden‐headed lion tamarins *Leontopithecus chrysomelas* – remains limited. As these primates spend up to half of their daily activity budget foraging in the wild, food‐based enrichment may significantly improve welfare in captivity. Here, we investigated the effects of food puzzles on six zoo‐housed golden‐headed lion tamarins (three pairs) over 17 days (5 control, 12 treatment). Food engagement time and behaviours that may reflect stress or arousal states (grooming and activity) were recorded during structured observation periods. We found that food puzzles significantly increased feeding engagement, from an average of 15 min with standard food bowls to 47 min with puzzles (~3‐fold increase). In contrast, no statistically significant changes were detected in grooming or activity levels. Although short‐term exposure did not reduce stress‐related behaviours, the more equitable distribution of allogrooming and the decrease in self‐grooming observed in most individuals suggest potential group‐level benefits. Our findings underscore the potential benefits of food puzzles which reliably extend foraging time in captive golden‐headed lion tamarins. Our work also highlights the need for longitudinal, multi‐zoo studies to evaluate effects on social dynamics and welfare.

## Graphical abstract

Food puzzles significantly increased food engagement time in captive golden‐headed lion tamarins (*Leontopithecus chrysomelas*), with individuals spending three times longer foraging compared to standard food bowls. Grooming and activity levels showed no short‐term changes, though patterns varied across enclosures. These findings highlight puzzle feeders as effective enrichment for promoting natural foraging behaviours in small‐bodied primates, while underscoring the need for longer‐term studies to assess effects on stress and social dynamics.

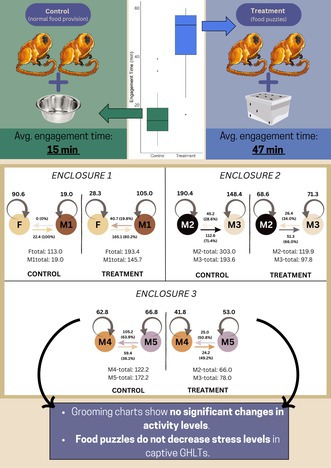

## 1 Introduction

Environmental enrichment, particularly food‐based puzzles, is widely recognised as an effective strategy to reduce stress and promote natural behaviours in captive primates (Reinhardt 1994; Lee et al. 2008; Clark 2011; Sanders and Fernandez 2022). Despite this, few studies have investigated enrichment in golden‐headed lion tamarins ( *Leontopithecus chrysomelas* ; GHLTs). These callitrichids are naturally organised in small, cohesive social groups composed of related individuals, which explains why the species is typically housed in smaller groups and enclosures in captivity (Hankerson et al. 2006). In contrast, larger primate species exhibit more variable social structures, including both small familiar groups and larger multi‐male/multi‐female groups, meaning small housing for these species may not reflect their natural social organisation (Farmer et al. 2023; Brando et al. 2023). While callitrichids in research laboratories have been the focus of multiple enrichment studies (Buchanan‐Smith 2011; Sha et al. 2016; Wissman 2014), zoo‐housed callitrichids remain under‐studied, leaving important gaps in our understanding of the effects of environmental enrichment on these individuals.

Environmental enrichment not only alters behaviour but is also closely linked to physiological welfare in captive primates. The introduction of enrichment strategies has, for example, been shown to decreases plasma cortisol in brown capuchins (Boinski et al. 1999) and change the microbiota in mice and rabbits (Feng et al. 2022; Lupori et al. 2022). These physiological indicators illustrate that enrichment impacts are multi‐dimensional, affecting both observable behaviour and underlying physiology.

Many animals, particularly vertebrates, are healthier when provided environments that support a full repertoire of natural behaviours. This relationship has been most extensively demonstrated in mammals and birds housed in captive or managed environments (Mason 1991). In the wild, callitrichids spend up to half of their daily activity budget foraging (Wormell et al. 1996; Garber 1993). In captivity, the opportunity to forage is limited, which can contribute to stress (Lutz and Novak 2005). For GHLTs, food puzzles are a promising form of enrichment because they mimic natural micromanipulation behaviours, where individuals use elongated hands and fingers, i.e., relatively long, slender digits adapted for probing and extracting small food items from crevices, to extract embedded food (Rapaport 1998; Wormell et al. 1996; Rylands 1989; Stoinski et al. 2003). However, this trait is not universal across callitrichids: for example, cotton‐top tamarins ( *Saguinus oedipus* ) are more reluctant to reach into cavities, illustrating the importance of species‐specific puzzle design.

In addition to foraging, grooming is a key indicator of welfare in primates. It promotes relaxation, positive emotional states, and social bonding (Schino et al. 1988; Dunbar 1991, 2010, 2013; Ventura et al. 2006; Russell and Phelps 2013). In callitrichids, which are highly social cooperative breeders (Goldizen 1990; Mustoe 2023), grooming represents an important component of group cohesion (Löttker et al. 2007). Effective enrichment strategies for primates are often associated with changes in grooming and activity patterns, including increased grooming and reduced overall activity (Chamove and Moodie 1990; Costa et al. 2018), but to date, this has not been demonstrated in GHLTs. This is significant since enrichment research is important within a conservation context (Cade 1986), and the GHLT is indeed an endangered species (Oliveira et al. 2021). Captive‐born tamarins destined for reintroduction often have lower survival rates than their wild‐born counterparts, partly due to underdeveloped foraging and locomotion skills (Kierulff et al. 2012; Stoinski and Beck 2004). High stress levels also impair reproductive success in callitrichids, threatening the sustainability of captive breeding programmes (Wormell et al. 1996; Honess and Marin 2006). Enrichment that extends foraging time and promotes natural behaviours can therefore contribute directly to both welfare and conservation outcomes.

The effects of environmental enrichment on captive primates can vary across species depending on ecology, social systems, and sensitivity to environmental change. Despite the close phylogenetic relationship between GHLTs and golden lion tamarins ( *Leontopithecus rosalia* ), empirical data on the welfare impacts of enrichment on GHLTs remain extremely limited. This represents an important gap, given the endangered status of the species and the reliance on ex situ populations for conservation management. Food‐based puzzle enrichment offers a non‐invasive approach to increasing foraging engagement while allowing animals to retain control over their interactions with the enrichment. Testing such enrichment in GHLTs is necessary to assess whether it promotes sustained food engagement without disrupting affiliative behaviours or increasing activity levels associated with heightened arousal. Here, we investigate whether food puzzles influence food engagement and behaviours commonly used as indirect indicators of stress or arousal in zoo‐housed GHLTs. Specifically, we test the prediction that food puzzles will (i) increase food engagement time, (ii) increase grooming and (iii) decrease activity. This predicted reduction in activity assumes that puzzles transition restless or aimless movement and stereotyped behaviours into more focused food manipulation. By addressing these questions, our study provides one of the first assessments of puzzle enrichment in this endangered species and contributes to the evidence base for best practice in callitrichid management and ex situ conservation.

## 2 Material and Methods

### 2.1 Study Site and Subjects

We used an ethogram to collect behavioural data (Table 1). Data collection took place from May 26 to June 14, 2023, at Tropikariet, a small indoor zoo in Helsingborg, Sweden. Data collection was necessarily limited in duration due to financial and time constraints. Three pairs of GHLTs were observed, each housed in a separate enclosure (see Table 2). Only the pair in Enclosure 1 was a male–female pair; the other two enclosures housed male–male pairs. Individuals were not re‐paired for the study; they were already permanently housed in this configuration. The animals were not on display to the public and were kept in an area of the zoo only accessible to staff members. Additional information on individuals is provided in Table 2. None of the pairs had visual access to one another during the trials.

**TABLE 1:** Ethogram of observed behaviours. Behaviours are either classified as ‘active’, ‘inactive’ or ‘grooming’.

Behaviour name	Description of behaviour	Classification
Jumping	GHLT jumps from one place to another	Active
Moving	GHLT moves around relatively slowly	Active
Running	GHLT runs/moves around speedily	Active
Eating	GHLT puts food in mouth, chews and swallows it	Active
Scratching	GHLT scratches himself/herself	Active
Biting	GHLT uses teeth to bite down on the other GHLT in its enclosure	Active
Ground	GHLT is located on the ground surface of their enclosure and is walking around/rummaging through the substrate	Active
Chasing	GHLT chases the other GHLT in their enclosure	Active
Fighting	GHLT has aggressive physical with the other GHLT in their enclosure (sequences of scratching/pulling/biting/chasing)	Active
Food bowl interaction	GHLT rummages through their food bowl or interacts with it in a different way (e.g., touching/smelling)	Active
Other food interaction	GHLT interacts with their food in a different way (e.g., chews food and spits it out, peels food with teeth, handles the food, inspects the food)	Active
Cage	GHLT interacts with the caged door of the enclose (e.g., climbs on it, hangs from it)	Active
Laying down	GHLT lays down on surfaces (sleeping boxes/branches/other) and remains laid down in the same place	Inactive
Sitting	GHLT sits down on surface (sleeping boxes/branches/other) and remains seated in the same place	Inactive
Sleeping	GHLT is stationary (sitting/laid down) and has its eyes closed	Inactive
Out of sight	GHLT is stationary in their sleeping box/tree trunk and cannot be seen by the observer	Inactive
Self‐grooming	GHLT grooming directed at own body	Grooming
Allogrooming	GHLT grooming directed at other GHLT	Grooming

**TABLE 2:** Information about research subjects. Column ‘Time spent backstage’ reflects how long the individuals have been housed away from visitors and their family group, also housed at Tropikariet. ‘Time spent together’ reflects how long the pairs had been housed together at the time data was collected. Ages and periods of time are recorded in years (Y) and months (M).

Enclosure	Size	Individual	Age	Time spent backstage	Castrated	Time since group formation
1	320 × 120 × 270 cm	Female (F)	3 Y, 0 M	2 Y, 8 M	No	2 Y 6 M
		Male1 (M1)	4 Y, 1 M	2 Y, 7 M	Yes	
2	310 × 320 × 270 cm	Male 2 (M2)	4 Y, 11 M	1 Y, 4 M	No	1 Y 4 M
		Male 3 (M3)	3 Y, 5 M	2 Y, 6 M	No	
3	370 × 330 × 270 cm	Male 4 (M4)	3 Y, 0 M	6 M	No	6 M
		Male 5 (M5)	2 Y, 3 M	7 M	No	

### 2.2 Study Design

The study spanned 17 days: five control days, during which food was provided in bowls, and 12 treatment days, during which food was provided in puzzle feeders. Each day included two observation periods per enclosure: one in the morning (‘food observations’) and one in the afternoon (‘stress observations’). On control days, morning observations coincided with standard food bowl provision. During treatment, the same diet and quantities were presented in puzzle feeders, which replaced the food bowls (ca. 120–150 g per group, following the European Association of Zoos and Aquaria's (EAZA) best practice guidelines for callitrichids; European Association of Zoos and Aquaria (EAZA), n.d.). After puzzle observations ended, remaining food was transferred to the animals' bowls to ensure they received their full ration. Puzzle devices were needed for subsequent trials and the removal of the boxes ensured similar exposure times in all groups. Afternoon feedings took place after stress observations. The observation schedule was fixed, with enclosures observed in the same order each day (Table S1). Food puzzles were deployed by the observer at the start of morning trials.

### 2.3 Diet and Food Puzzles

The tamarins' diet followed EAZA guidelines (European Association of Zoos and Aquaria (EAZA), n.d.) and consisted of 40% root vegetables (2–3 varieties every day of e.g., potatoes, carrots, beets, parsnips), 30%–40% other vegetables (4–5 varieties every day), 10%–15% protein (mostly insects, such as zoophobas, but also including boiled eggs or boiled chicken hearts), and 5%–10% fruits. EAZA recommends the removal of fruit from callitrichid diets, and Tropikariet was actively working towards achieving this goal, although this was not yet reached at the time this study took place. The GHLTs were also provisioned with new world pellets (ad libitum), although this was not considered as a part of this study. Puzzle feeders were made from transparent 15 L plastic boxes with lids (39 × 29 × 20 cm; Figure S1). Each box had 15 holes (four per long side, seven in the lid), their edges taped for safety. Two internal dividers created side compartments accessible through the holes. To encourage manipulation, three plastic shot glasses and sections of plastic garden fencing were placed in the central compartment. Food was distributed across compartments so that items were embedded and partially concealed. The transparent design allowed animals to see the food and facilitated navigation. GHLTs were fed again later in the afternoon, although these feeding events were not a part of our study. The subjects had been exposed to simpler puzzle feeders prior to the study, though not for at least 4 weeks. This previous experience may have influenced responses and is considered in the discussion.

### 2.4 Data Collection

All observations were recorded via low‐volume voice notes on a mobile device (Bateson and Martin 2021), later transcribed into behavioural data. We carried out continuous sampling (Altmann 1974), where every behavioural transition was timestamped; we calculated the number of seconds spent on each behaviour using timestamps. Morning food observations took place between 9:00 and 11:45, lasting until both individuals in a pair had ceased feeding or puzzle manipulation for at least 10 min, or until 1 h had elapsed. Afternoon stress observations began exactly 3 h later and lasted 30 min per pair. Callitrichid digestive transit times are relatively quick, with most food moving through the gut within ~3 h in related species, suggesting that behaviour assessed after this interval would not be strongly influenced by ongoing digestion (Lapenta and Procópio‐de‐Oliveira 2008). Data were collected using focal pair sampling. Variables recorded included total food engagement time (minutes), grooming (self and allogrooming, minutes), inactivity (minutes) and activity (minutes). Foraging was classified as ‘activity’ during afternoon stress observations because it was largely absent at that time point. We observed that foraging was restricted to a brief period immediately following food provision in both control and treatment periods, with almost no foraging behaviour occurring during the afternoon stress observations conducted 3 h post food provision. Consequently, foraging contributed negligibly to the behavioural data collected during these sessions. When foraging did occur, it rarely resulted in consumption and was considered potentially abnormal or boredom‐related (Table S1). For analytical consistency, foraging was therefore grouped within the broader category of ‘activity’. This definition differs from broader welfare practice, where activity is not inherently negative, and is discussed as a limitation of the current design.

### 2.5 Enclosure Characteristics

Enclosure 1 was smaller, housed a female–castrated male pair and was located nearest staff walkways and the kitchen, resulting in the highest relative human traffic with an average of 8 people walking by during the second round of focal pair sampling on treatment days, as opposed to enclosures 2 and 3 with an average of approximately 7.33 and 0.77. Enclosures 2 and 3 housed male pairs in larger spaces with less visitor exposure. These visitor traffic values were used as averages to help interpret potential confounding factors but were not the focus of our study.

### 2.6 Behavioural Definitions

Behaviours were classified into three categories: active, inactive and grooming (see Table 1). We grouped all behaviours, including state behaviours (e.g., sleeping, moving) for further analyses. Grooming was defined as deliberate manipulation of hair or skin using hands or mouth (Person 1998). Grooming directed at one's own body was considered self‐grooming; grooming directed at a partner was considered allogrooming. Grooming was analysed both as a combined measure and as separate behaviours.

### 2.7 Data and Analysis

The final data set comprised 17 days of observation (5 control, 12 treatment), generating 102 focal periods (51 food observations and 51 stress observations). Each entry included treatment type, study day, enclosure, individual identity and time spent in each behavioural category. Due to a fire alarm, data from the twelfth treatment day were excluded. Our analyses tested for differences between control and treatment periods without modelling temporal trends to avoid autocorrelation. Statistical analyses were conducted in R (R Core Team 2024) using RStudio (RStudio Team 2022).

*Food engagement time* was analysed using a generalised linear model (GLM) and modelled using treatment as a predictor and fixed effect, with a Gamma error distribution and log link in base R to account for positively skewed continuous data. Enclosure was initially included as a random effect; however, the model resulted in a singular fit with the random‐effect variance estimated as zero, indicating no detectable variation attributable to enclosure. Enclosure was therefore excluded from the final models and data were combined for analysis across enclosures to improve statistical power. Final inference was based on the GLM without random effects.

*Activity levels* were converted into proportional rates (decimal form) using the ‘dplyr’ package (Wickham et al. 2023) and analysed using a linear mixed‐effects model (LMM) fitted by restricted maximum likelihood (REML) using the lmer function in the lme4 package (Bates et al. 2015). *p*‐values were calculated using lmerTest (Kuznetsova et al. 2017). A Gaussian distribution was assumed. The response variable were the rates and the activity type (Activity, Inactivity, Grooming), treatment and their interaction were included as fixed effects. Individual identity was included as a random effect to account for repeated measures within individuals. Enclosure was initially considered as an additional random effect but was removed following likelihood ratio testing, as it explained no detectable variance. Degrees of freedom and *p*‐values for fixed effects were estimates using Satterthwaite's approximation as implemented in the lmerTest package (Kuznetsova et al. 2017). Model assumptions were assessed by inspection of residual and quantile‐quantile plots.

*Grooming behaviour* was analysed separately for self‐grooming and allogrooming using LMMs. The lmer function in the lme4 package (Bates et al. 2015) was used, with *p*‐values from lmerTest (Kuznetsova et al. 2017) and treatment was added as a predictor. The duration of each behaviour (in seconds) was square‐root‐transformed to stabilise variance. To account for repeated measurements, individual identity was included as a random effect in both models. Preliminary analyses showed negligible variation among enclosures, so enclosures were combined for analysis to improve statistical power. Model assumptions were evaluated via residual plots and histograms of scaled residuals.

Data visualisation was performed in *ggplot2* (version 3.4.1; Wickham 2016). Grooming data were also presented graphically using Canva (Canva.com).

## 3 Results

### 3.1 Food Engagement

**FIGURE 1:**
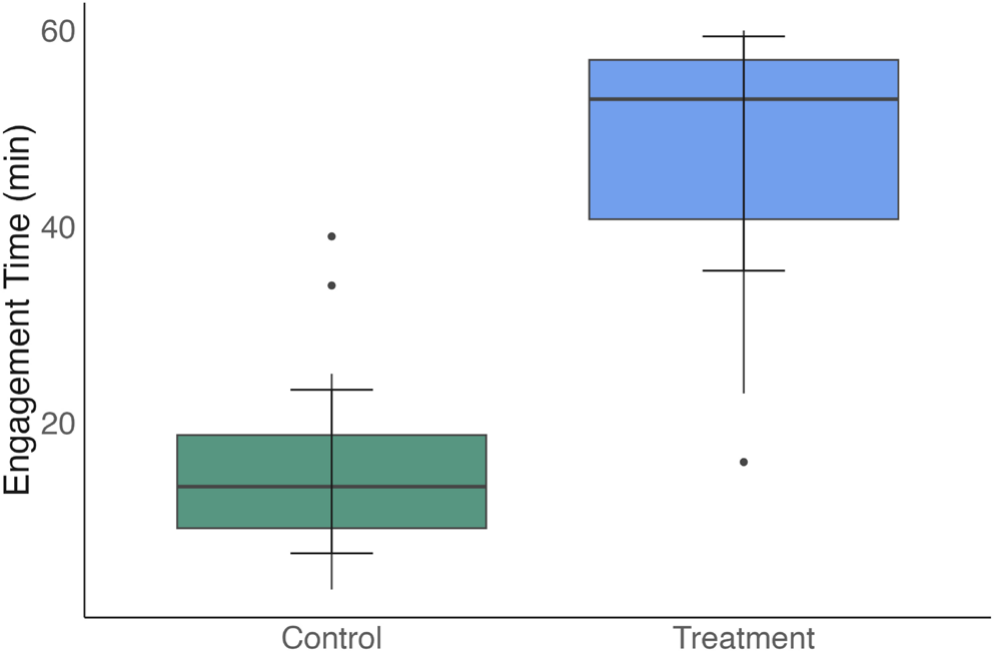
Food engagement time by treatment. Food engagement time in Control and Treatment periods. Boxplots show the median, quartiles and whiskers (1.5 × IQR). Error bars represent 1 standard deviation from the mean. A generalised linear model with Gamma distribution and log link indicated that Treatment significantly increased engagement time relative to Control (*B* = 1.15 ≤ 0.08, *p* < 0.001), corresponding to a 3.2‐fold increase.

Provision of food puzzles significantly increased food engagement time (Gamma GLM, likelihood ratio *χ* ^2^_1_ = 23.24, *p* < 0.001; Figure 1). Individuals engaged with food bowls for an average of 15 min on control days, whereas engagement with puzzle feeders increased to an average of 47 min on treatment days. Our GLM revealed a strong positive effect of puzzle provision on engagement time (*β* = 1.15 ± 0.08 SE, *p* < 0.001), corresponding to a 3.16‐fold increase in engagement relative to control conditions (exp[*β*] = 3.16).

### 3.2 Activity and Grooming

**FIGURE 2:**
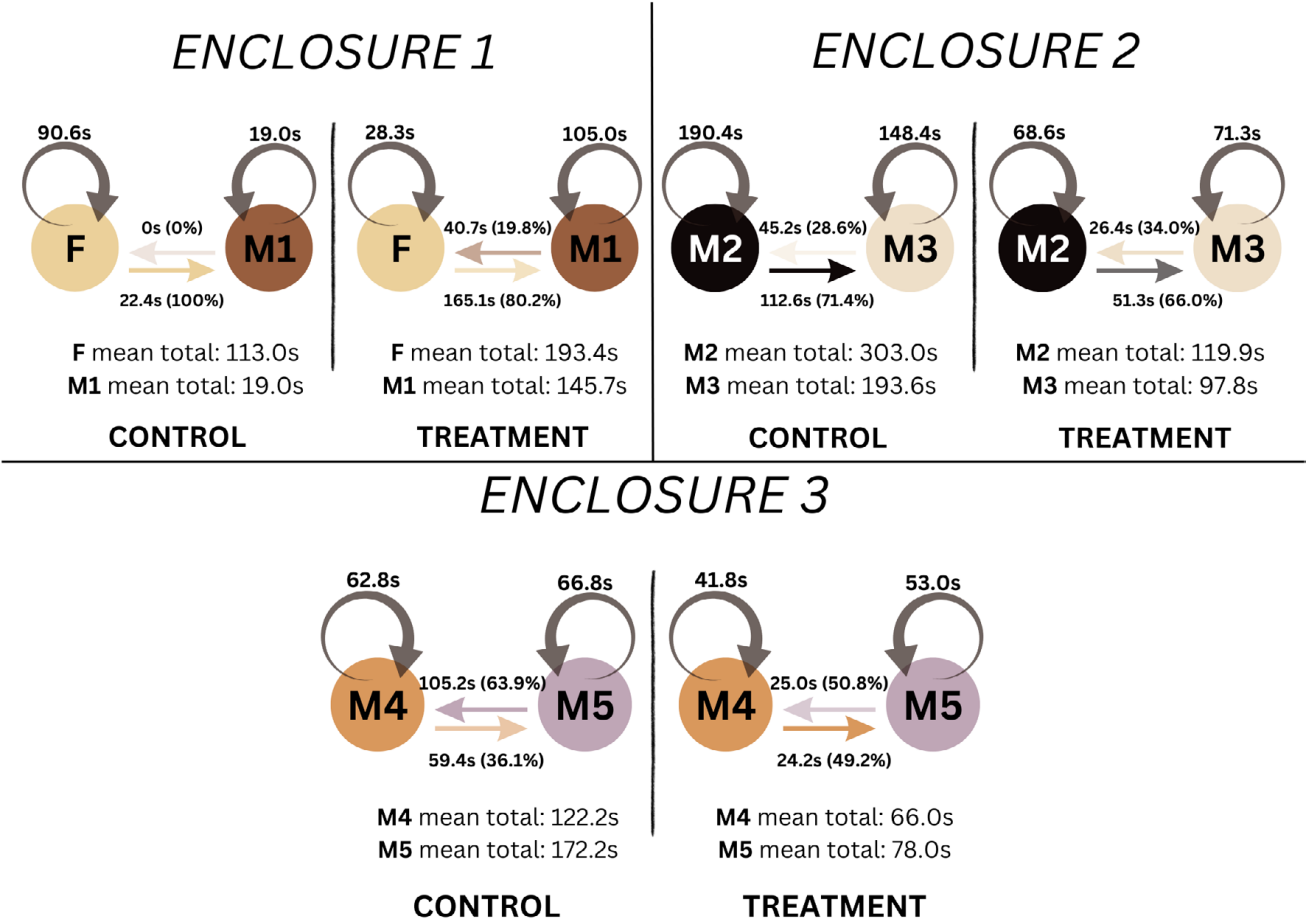
Grooming charts per enclosure. Three grooming charts reflecting mean grooming times and rates across control and treatment periods (one per enclosure). Circular arrows and their corresponding numbers reflect the mean self‐grooming activity of that individual in seconds. Horizontal arrows reflect mean allogrooming activity per individual in seconds and percentages. The arrow points from the GHLT that performed the grooming to the GHLT that received the grooming. The mean total length of time individuals spent grooming (self‐grooming + allogrooming) (in seconds) is included at the bottom of each chart. All variables are reported for both control and treatment periods.

Stress‐related behaviours were evaluated during afternoon observations. Treatment had no effect on activity allocation (main effect of treatment: *F*_1,300_ = 0.00, *p* = 1.00, Figure 2), and there was no evidence that treatment altered the relative distribution of time across activity categories (Type × Treatment interaction: *F*_2,300_ = 0.28, *p* = 0.75). Thus, activity budgets did not differ between control and treatment conditions. Our self‐grooming model indicated that treatment tended to reduce self‐grooming, although it was not statistically significant (*β* = −1.72 ± 1.34, *t* = −1.28, *p* = 0.20). Individual differences contributed negligible variance, indicating that repeated measures had minimal influence on self‐grooming. For allogrooming, treatment had no detectable effect (*β* = −0.48 ± 1.29, *t* = −0.37, *p* = 0.71, Figure 2). Individual differences contributed modest variance, but overall treatment did not significantly affect allogrooming. Although group‐level effects were not statistically significant, enclosure‐level patterns suggested qualitative differences (Figure 2). Total grooming time increased in enclosure 1 but decreased in enclosures 2 and 3. Food puzzle treatment also led to more balanced allogrooming contributions between partners. For example, the female in enclosure 1 groomed exclusively pre‐treatment (100% of grooming time), but grooming became more evenly distributed post‐treatment (80% vs. 20% with the male partner). Self‐grooming decreased across all individuals except the castrated male in enclosure 1, where it increased.

## 4 Discussion

We predicted that food puzzles would increase food engagement time, increase grooming and decrease activity. Our results partially supported these predictions. As expected, food puzzles significantly increased food engagement time, with individuals spending approximately three times longer interacting with puzzles compared to food bowls. However, no statistically significant effects of treatment were found on grooming or activity budgets (Figure S2).

The strong effect of puzzles on food engagement is consistent with enrichment studies in other primates. For example, marmosets increased feeding time from 4 to 6 min to nearly 3 h after introduction of a food dispenser (Voelkl et al. 2001), and rhesus macaques increased puzzle feeder interaction by over 2000% (Reinhardt 1994). Similar increases have been reported in great apes (Clark 2011; Gottlieb et al. 2011; Lee et al. 2008; Gronqvist et al. 2013). Our findings extend this pattern to golden‐headed lion tamarins (GHLTs), for which enrichment evidence has been scarce. Importantly, puzzle feeders elicited prolonged foraging behaviour despite animals' prior experience with simpler devices, suggesting that even familiar enrichment can provide meaningful stimulation. In order to further promote adaptive behaviours, the authors recommend the transition from transparent to non‐transparent feeding boxes after the first few weeks of exposure to the enrichment devices.

Contrary to our predictions, grooming and activity did not change significantly across treatments (Figures 2 and S3). Although these behaviours are frequently used as indirect indicators of stress or arousal in primates, they are context‐dependent and do not exclusively reflect stress. In the present study, self‐grooming decreased in all individuals except for the castrated male in Enclosure 1, although these results were not statistically significant. Given that self‐grooming is sometimes associated with stress, this decline in most individuals may be interpreted as a potential positive outcome, though self‐grooming was not treated as abnormal in the present study. Although self‐grooming is sometimes associated with stress or displacement behaviour, its functional significance is context‐dependent and may also reflect self‐soothing, hygiene, or routine maintenance (McGlone et al. 2016). Consequently, the observed reduction in self‐grooming cannot be interpreted unambiguously as a positive welfare outcome. Instead, these changes may reflect shifts in time allocation, behavioural priorities, or social context associated with increased engagement with enrichment. The observed changes in grooming behaviour highlight the complexity of interpreting affiliative and self‐directed behaviours and underscore the need for caution when inferring welfare states from grooming patterns alone.

The shifts we observed in allogrooming patterns may reflect changes in social dynamics rather than simple changes in stress levels. In cooperative breeding callitrichids, grooming plays an important role in maintaining social cohesion, reinforcing affiliative bonds and negotiating social roles (Dunbar 1991; Lazaro‐Perea et al. 2004), and a more equitable distribution of grooming may indicate altered patterns of social engagement within the pair. Increases in grooming and reductions in activity have been reported following enrichment in some species, including cotton‐top tamarins, white‐handed gibbons, mona monkeys and brown lemurs (Chamove and Moodie 1990; Costa et al. 2018). However, other studies have found negligible or inconsistent effects, particularly in rhesus monkeys, Javan gibbons and chimpanzees (Lee et al. 2008; Gronqvist et al. 2013; Padrell et al. 2022). Thus, the absence of an effect in our study may reflect both species‐specific differences and the limited timeframe. Short‐term enrichment may not be sufficient to alter behavioural time budgets, particularly in highly social callitrichids, where affiliative patterns develop over longer periods.

While our models did not detect significant enclosure effects, qualitative differences and patterns of grooming were observed to vary with enclosure context. Grooming increased in the female–castrated male pair (enclosure 1) but decreased in male–male pairs (enclosures 2 and 3). Enclosure 1 was smaller and more exposed to visitors, but this is unlikely to alone generate the patterns found here (Figures S4–S6). Although these observations likely reflect the specific social compositions and physical contexts of these individuals rather than broader trends for captive populations, these contextual influences underscore the importance of considering enclosure characteristics and social composition when evaluating enrichment outcomes.

In this study, activity during afternoon observations was classified as potentially stress‐related as these behaviours rarely resulted in food consumption and were interpreted as indicative of restlessness or boredom. We acknowledge, however, that this approach conflates distinct behavioural types and may obscure important differences: for example, some activity may represent positive exploration or play, while grooming is primarily a social behaviour. Grouping behaviours in this way is a limitation of our study design and may mask the independent contributions of social, exploratory and displacement behaviours to welfare outcomes. Future studies should consider distinguishing these categories more precisely, e.g., separating functional activity, social interactions and potential abnormal behaviours (e.g., stereotypies) to more accurately evaluate enrichment effects.

It is worth noting that our results should be interpreted with caution due to limitations. First, the study was short‐term (17 days), which may explain why no clear effects on stress‐related behaviours were detected. Longitudinal designs are needed to assess whether extended exposure leads to sustained changes in grooming, activity, or abnormal behaviours. Second, although the animals had prior experience with enrichment, we directly observed them for 4 weeks immediately preceding the study and did not record any puzzle exposure. Therefore, while the puzzles were novel during the study period, prior experience may still have influenced their responses. Future work could compare naïve and experienced groups to clarify the role of novelty. Third, the study involved a small number of individuals and a limited number of social pairs; we acknowledge that social context can influence interactions and the restricted sample may limit the generalizability of our findings. Consequently, the lack of significant effects should be interpreted with caution, as the limited sample size may have resulted in insufficient statistical power to detect subtle behavioural changes. Fourth, variation in visitor traffic and enclosure characteristics likely contributed to behavioural differences. These factors should be explicitly incorporated as covariates in larger, multi‐zoo studies. Finally, we did not collect post‐treatment data, which limits our ability to evaluate persistence of effects once enrichment was removed. A follow‐up phase would be critical to determine whether benefits extend beyond immediate exposure.

Despite these limitations, our findings highlight the potential of puzzle feeders to promote natural foraging behaviours in GHLTs. Extended food engagement may improve welfare by reducing inactivity and providing cognitive stimulation, both of which are important for the development of foraging and locomotion skills. Such skills are critical for successful reintroduction. Evidence from a closely related species, the golden lion tamarin, indicates that zoo‐born individuals show lower survival rates compared to their wild‐born counterparts (Kierulff et al. 2012; Sanders and Fernandez 2022). Incorporating enrichment that reliably prolongs foraging could therefore strengthen both welfare practices in zoos and ex situ conservation outcomes. Although *L. rosalia* is a different species from the GHLT, both are callitrichids with broadly similar ecology, foraging strategies and reintroduction challenges. Nevertheless, species‐specific differences may influence reintroduction outcomes, and direct data on GHLTs are needed to confirm the relevance of these patterns.

## 5 Conclusions

Food puzzles appear to be a practical and effective means of extending food engagement in captive GHLTs. While our short‐term study did not demonstrate significant changes in grooming or activity, food puzzles consistently increased foraging behaviour. These results demonstrate that puzzle enrichment can effectively extend food engagement in zoo‐housed GHLTs, but do not provide evidence for broader effects on other behavioural measures. Longer‐term and larger‐scale studies are therefore required to evaluate whether extended exposure to puzzle feeders influences other aspects of welfare. We recommend that future studies extend observation periods, incorporate physiological measures of stress (e.g., cortisol) and assess post‐treatment effects to fully assess the long‐term impact of puzzle feeders on welfare and reintroduction potential.

## Author Contributions

**Giulia Pipolo:** conceptualization (equal), data curation (lead), formal analysis (lead), investigation (lead), methodology (lead), visualization (equal), writing – original draft (lead), writing – review and editing (equal). **Emma Chen:** data curation (supporting), formal analysis (supporting), methodology (supporting), writing – original draft (supporting). **Elin Harlos:** investigation (supporting), methodology (supporting), writing – original draft (supporting). **Johnny Möllerstrom:** conceptualization (supporting), investigation (supporting), methodology (supporting), writing – original draft (supporting). **Juliano Morimoto:** conceptualization (equal), data curation (supporting), formal analysis (supporting), funding acquisition (supporting), methodology (supporting), project administration (lead), supervision (lead), visualization (equal), writing – original draft (equal), writing – review and editing (lead).

## Funding

G.P. was supported by the E.W. Fenton Endowment Fund from the University of Aberdeen.

## Ethics Statement

This study was non‐invasive, and the protocol was reviewed and approved by the management at Tropikariet. According to Swedish law, enrichment studies like this one do not require ethical approval by Swedish authorities. Interaction with the food puzzle was entirely voluntary, subjects were provided with a normal diet, and normal management conditions were maintained throughout the study.

## Conflicts of Interest

The authors declare no conflicts of interest.

## Supporting information


**Data S1:** ece373312‐sup‐0001‐Supinfo.csv.

**Table S1:** Potential observation schedule, in which start‐ and end times of observations can be used as guidelines for future studies.
**Figure S1:** Pictures of food puzzle boxes. Sides of the boxes (39 cm) have four holes of the same size, while lids of the boxes have seven holes of the same size. Food is dispersed across both side sections of the puzzles (accessible through the holes in the sides), and the bigger middle section (accessed through the holes in the lid). Flexible garden fence and plastic shot glasses (3×) were placed in the middle section.
**Figure S2:** Average food engagement time across all enclosures over the span of our control and treatment periods. The blue dashed line reflects the average food engagement time during our control period (15 min) and the green dashed line reflects the average food engagement time during our treatment period (47.5 min). Red dotted line represents the separation between control and treatment periods.
**Figure S3:** Average activity levels (here defined as the average proportion of time, in percentages, individuals spent active and grooming) across all enclosures. Coloured lines reflect the average activity levels within each enclosure. Red dotted line represents the separation between control and treatment periods.

## Data Availability

Raw data were provided as Supporting Information.
