# Supplementary material for: Short‐Term Puzzle Feeder Enrichment Increases Food Engagement but Not Stress‐Related Behaviour in Captive Golden‐Headed Lion Tamarins
Source: Ecol Evol. 2026 Mar 27;16(4):e73312. doi: 10.1002/ece3.73312 (PMC13107288; doi:10.1002/ece3.73312)

**Supplementary material**

**Food puzzles increase food engagement time and can potentially improve group dynamics in captive golden-headed lion tamarins *Leontopithecus chrysomelas***

Authorship list: Giulia Pipolo^1^, Emma Chen^1^, Elin Harlos^2^, Johnny Möllerstrom^2^, Juliano Morimoto^3^

Authors’ affiliations:

^1^School of Biological Sciences, University of Aberdeen, Zoology Building, Tillydrone Ave, Aberdeen AB24 2TZ

^2^Tropikariet, Hävertgatan 21, Helsingborg 254 42, Sweden

^3^Institute of Mathematics, University of Aberdeen, King's College, Aberdeen AB24 3FX, UK

*Corresponding author: [juliano.morimoto@abdn.ac.uk](mailto:juliano.morimoto@abdn.ac.uk)

**
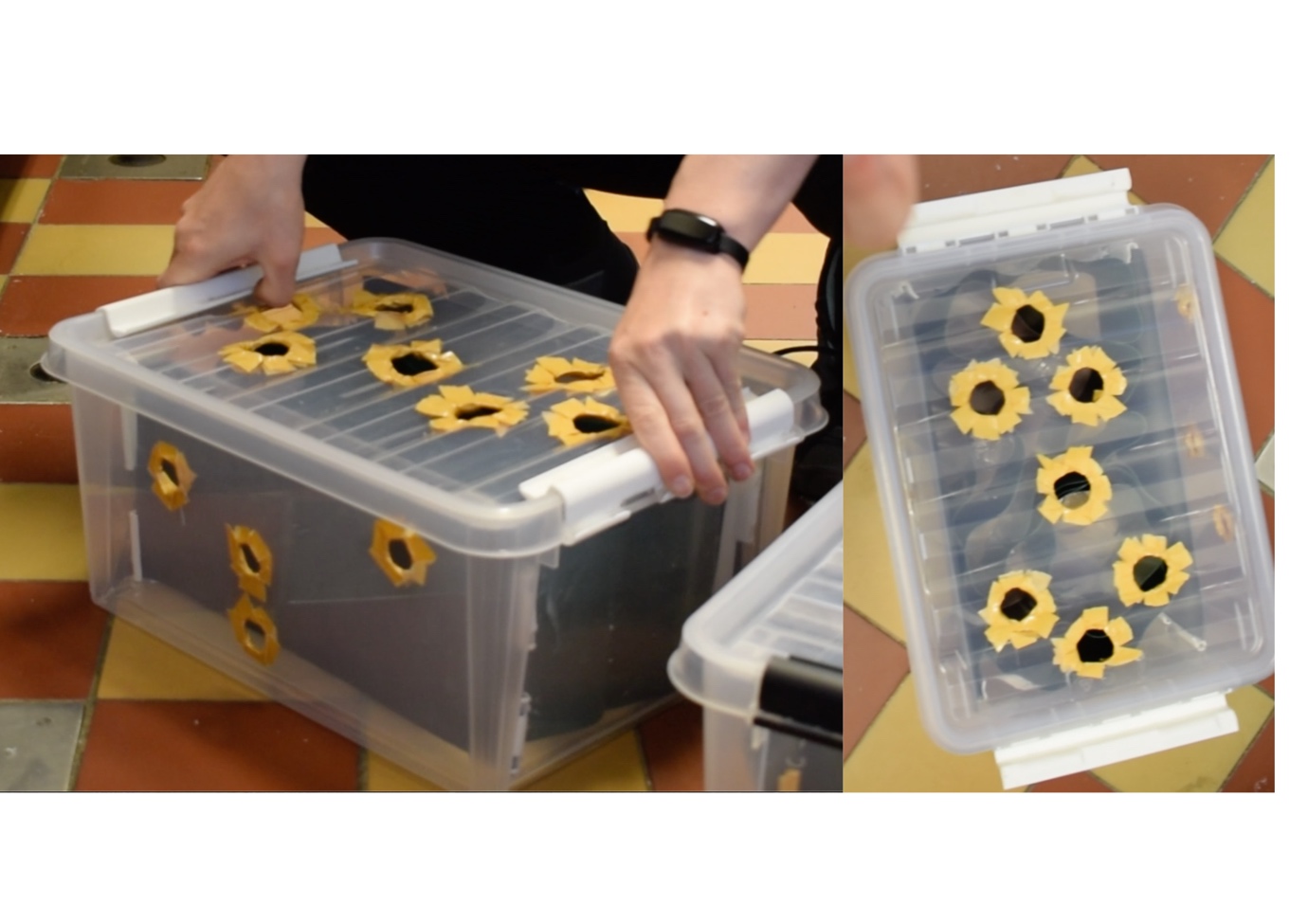
**

**Figure S1. Pictures of food puzzle boxes.** Sides of the boxes (39cm) have four holes of the same size, while lids of the boxes have seven holes of the same size. Food is dispersed across both side sections of the puzzles (accessible through the holes in the sides), and the bigger middle section (accessed through the holes in the lid). Flexible garden fence and plastic shot glasses (3x) were placed in the middle section.


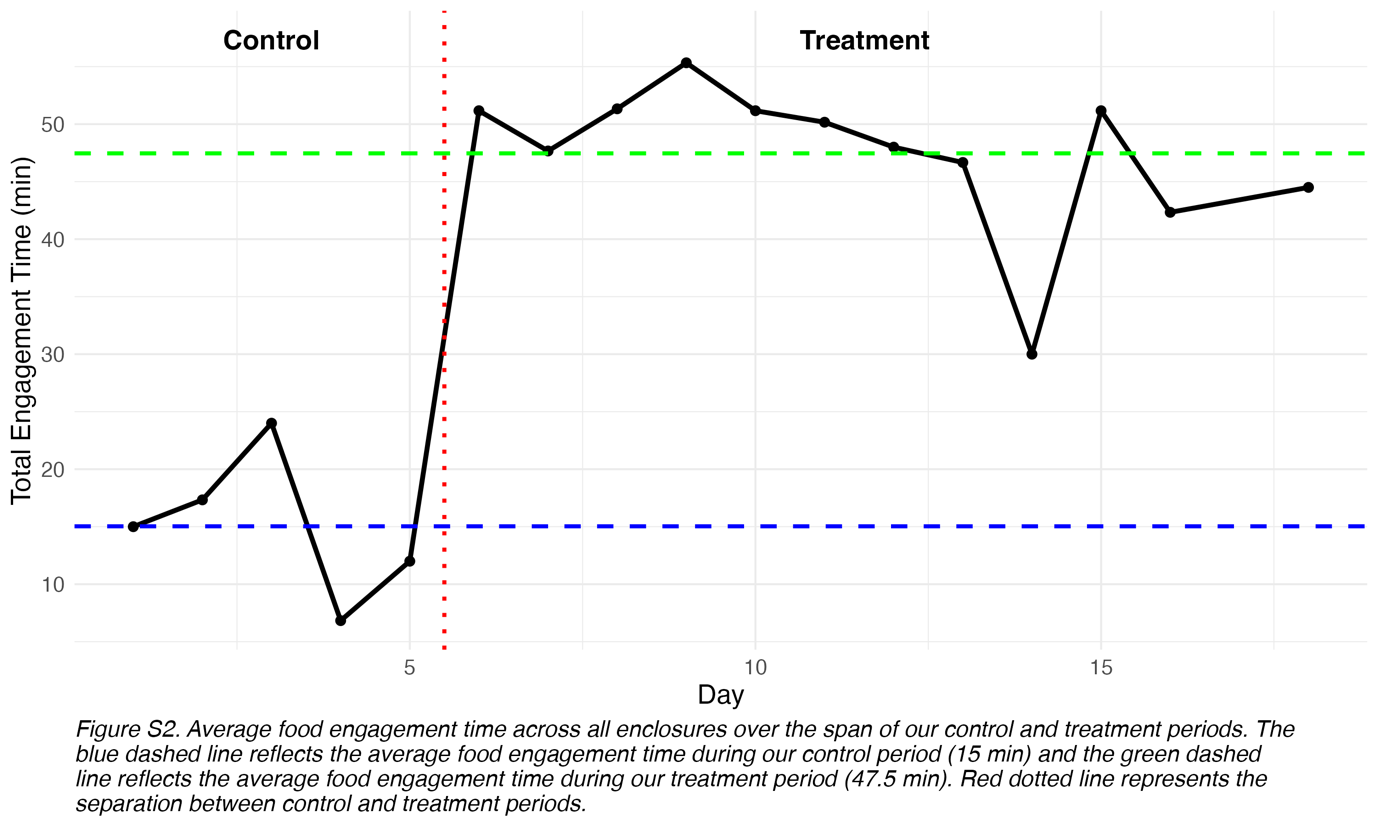


**Figure S2.** Average food engagement time across all enclosures over the span of our control and treatment periods. The blue dashed line reflects the average food engagement time during our control period (15 min) and the green dashed line reflects the average food engagement time during our treatment period (47.5 min). Red dotted line represents the separation between control and treatment periods.


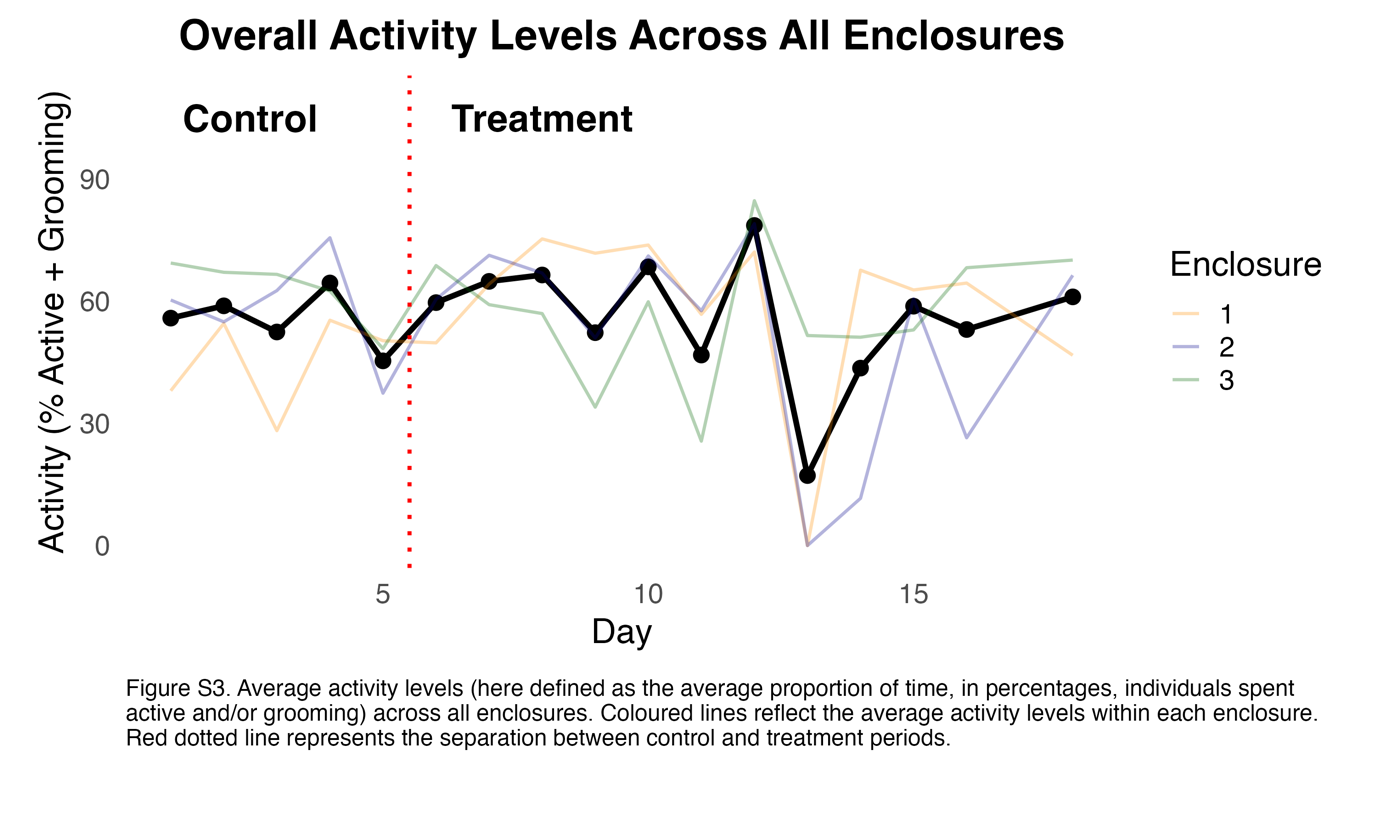


**Figure S3.** Average activity levels (here defined as the average proportion of time, in percentages, individuals spent active and grooming) across all enclosures. Coloured lines reflect the average activity levels within each enclosure. Red dotted line represents the separation between control and treatment periods.

**Table S1.** Potential observation schedule, in which start- and end times of observations can be used as guidelines for future studies.

| *Date* | *Observation type* | *Enclosure* | *Start time* | *End time* |
| --- | --- | --- | --- | --- |
| *Day 1(out of 5 control days)* | *Food observation (Control)* | *1* | *9:00* | *9:23* |
|  |  | *2* | *10:00* | *10:32* |
|  |  | *3* | *11:00* | *11:45* |
|  | *Stress observation (Control)* | *1* | *12:23* | *12:53* |
|  |  | *2* | *13:32* | *14:02* |
|  |  | *3* | *14:45* | *15:15* |
| *Day 1 (out of 12 treatment days)* | *Food puzzle observation (Treatment)* | *1* | *9:00* | *10:00* |
|  |  | *2* | *10:10* | *11:02* |
|  |  | *3* | *11:10* | *12:10* |
|  | *Stress observation (Treatment)* | *1* | *13:00* | *13:30* |
|  |  | *2* | *14:02* | *14:32* |
|  |  | *3* | *15:10* | *15:40* |


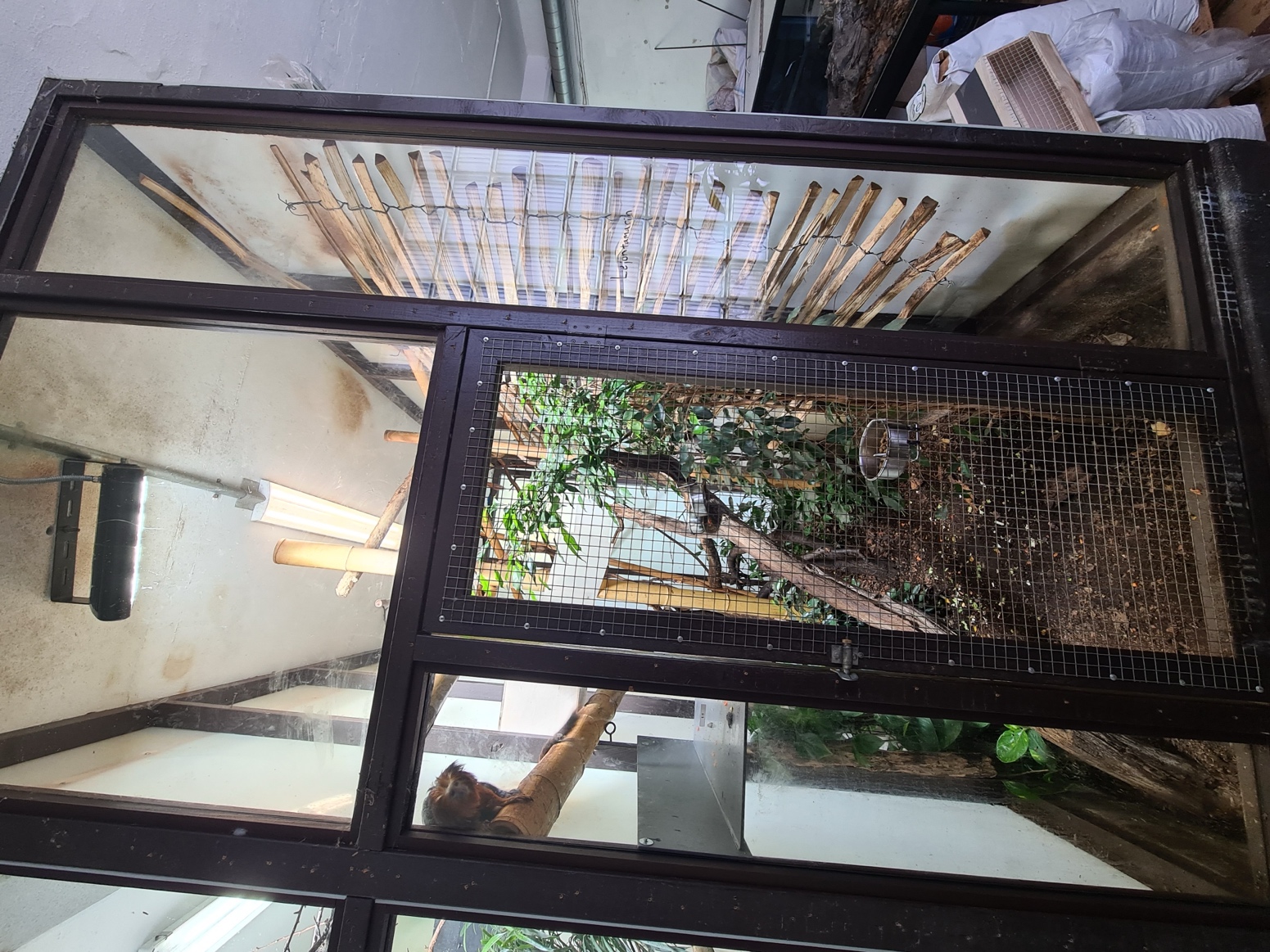


**Figure S4.** Picture of Enclosure 1.


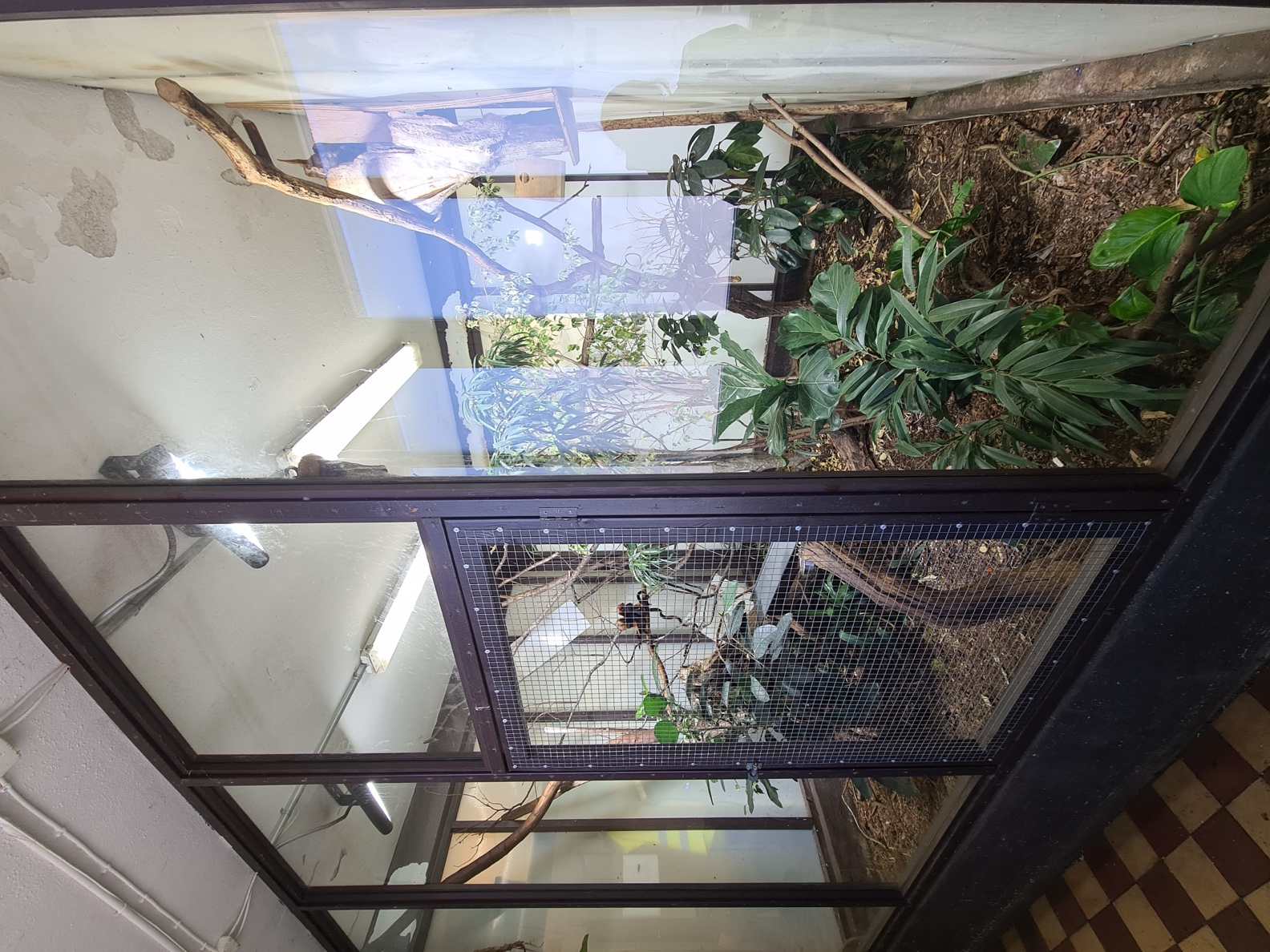


**Figure S5.** Picture of enclosure 2.


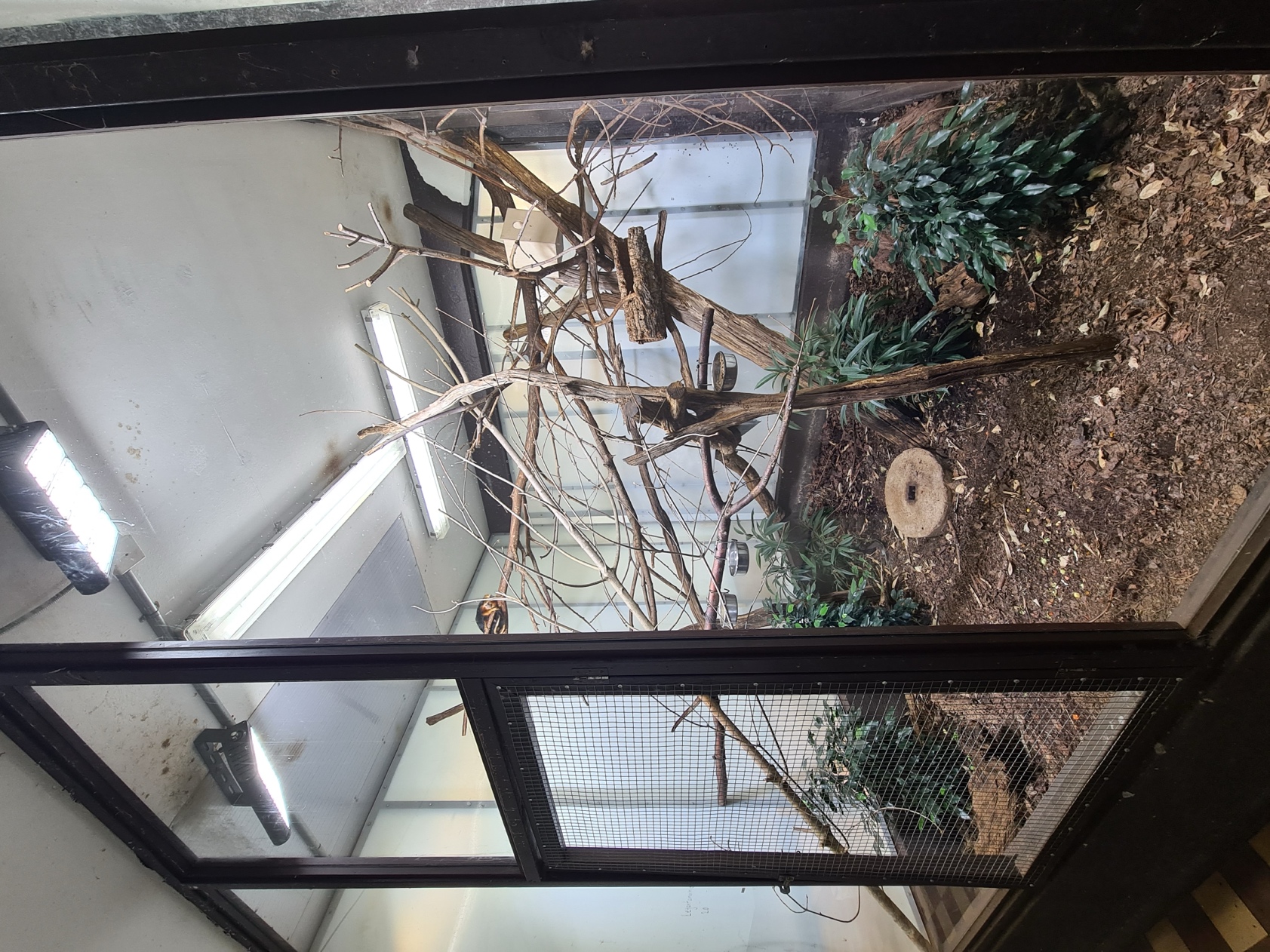


**Figure S6.** Picture of the enclosure 3.


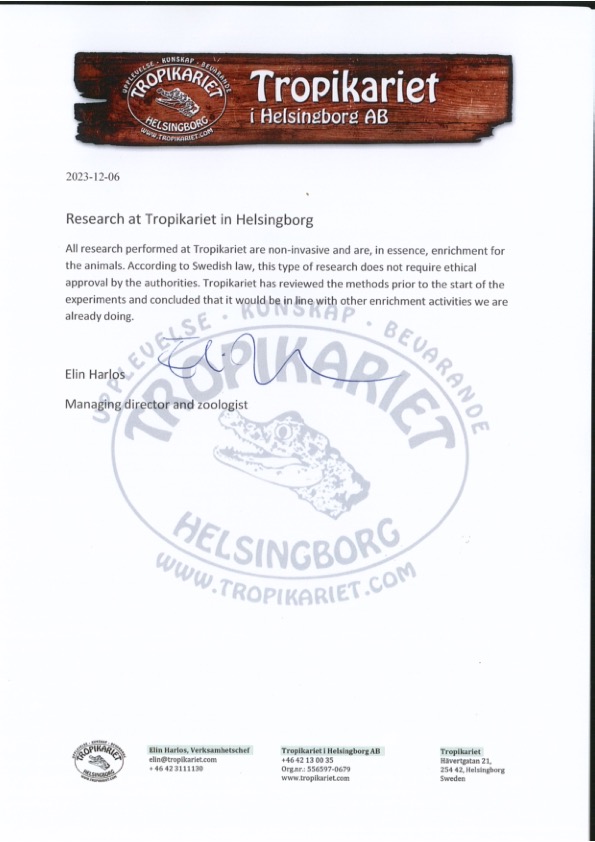

Supplement: Supplementary file 2 — Table S1: Potential observation schedule, in which start‐ and end times of observations can be used as guidelines for future studies. Figure S1: Pictures of food puzzle boxes. Sides of the boxes (39 cm) have four holes of the same size, while lids of the boxes have seven holes of the same size. Food is dispersed across both side sections of the puzzles (accessible through the holes in the sides), and the bigger middle section (accessed through the holes in the lid). Flexible garden fence and plastic shot glasses (3×) were placed in the middle section. Figure S2: Average food engagement time across all enclosures over the span of our control and treatment periods. The blue dashed line reflects the average food engagement time during our control period (15 min) and the green dashed line reflects the average food engagement time during our treatment period (47.5 min). Red dotted line represents the separation between control and treatment periods. Figure S3: Average activity levels (here defined as the average proportion of time, in percentages, individuals spent active and grooming) across all enclosures. Coloured lines reflect the average activity levels within each enclosure. Red dotted line represents the separation between control and treatment periods. [file ECE3-16-e73312-s001.docx]
